# Vitamin D and Sulforaphane Decrease Inflammatory Oxidative Stress and Restore the Markers of Epithelial Integrity in an In Vitro Model of Age-Related Macular Degeneration

**DOI:** 10.3390/ijms25126404

**Published:** 2024-06-10

**Authors:** Loredana Bergandi, Giulia Palladino, Alessandro Meduri, Laura De Luca, Francesca Silvagno

**Affiliations:** 1 Department of Oncology, University of Torino, Via Santena 5 bis, 10126 Torino, Italy; loredana.bergandi@unito.it (L.B.); giulia.palladino900@edu.unito.it (G.P.); 2 Ophthalmology Clinic, Department of Biomedical and Dental Sciences and Morphofunctional Imaging, University of Messina, 98125 Messina, Italy; alessandro.meduri@unime.it (A.M.); laura.deluca21@gmail.com (L.D.L.)

**Keywords:** vitamin D receptor, sulforaphane, transforming growth factor-β (TGF-β), human retinal pigment epithelial cells, inflammation, reactive oxygen species, vascular-endothelial growth factor (VEGF)

## Abstract

Age-related macular degeneration (AMD) is strictly linked to chronic oxidative stress, inflammation, loss of epithelial barrier integrity, and often with abnormal new blood vessel development. In this study, the retinal epithelial cell line ARPE-19 was treated with pro-inflammatory transforming growth factor-beta (TGF-β) to investigate the activity of vitamin D (VD) and sulforaphane (SF) in abating the consequences of oxidative stress and inflammation. The administration of VD and SF lowered reactive oxygen species (ROS) levels, and abated the related expression of the pro-inflammatory cytokines interleukin-6 and interleukin-8 induced by TGF-β. We evaluated mitochondrial respiration as a source of ROS production, and we discovered that the increased transcription of respiratory elements triggered by TGF-β was prevented by VD and SF. In this model of inflamed epithelium, the treatment with VD and SF also reduced the secretion of VEGF, a key angiogenic factor, and restored the markers of epithelial integrity. Remarkably, all the observed biological effects were potentiated by the co-stimulation with the two compounds and were not mediated by VD receptor expression but rather by the ERK 1/2 pathway. Altogether, the results of this study reveal the powerful synergistic anti-inflammatory activity of SF and VD and lay the foundation for future clinical assessment of their efficacy in AMD.

## 1. Introduction

Age-related macular degeneration (AMD) is a progressive eye disease affecting the macula, a highly specialized region of the retina that is responsible for central vision and perceiving fine details [1]. It has been reported to affect one in eight people aged over 60 years old, and it is projected, according to the current life expectancy, that its frequency will increase due to the aging population in Western countries as it represents the most common cause of irreversible central blindness in older people in developed countries [2]. The etiology of AMD is poorly understood although it has been linked to several pathological factors, i.e., chronic oxidative stress, autophagy decline, and inflammation [3]. It occurs in two forms, namely, as dry (atrophic) or wet (exudative, neovascular) AMD [4]. The retina is consistently bathed in an oxidative environment because of its high oxygen consumption, high polyunsaturated fatty acid (PUFA) content, and exposure to light [5]. With age, oxidative damage increases and antioxidant capacity decreases, as does the efficiency of reparative systems [6], leading to tissue dysfunction. Thus, a substantial contributor to macular degeneration is oxidative stress, which refers to cellular damage caused by reactive oxygen species (ROS), sustaining the perpetuating inflammation due to the excessive or inappropriate long-term oxidative insult [7,8], a process that has been implicated in both advanced forms of AMD [5,9]. In dry AMD, aging leads to chronic senescent and a decrease in the density of the human retinal pigment epithelium (hRPE) cells with altered mitochondrial metabolism, accompanied by increased drusen deposition, overlying photoreceptor dysfunction and atrophy, and/or neovascularization [10,11]. Indeed, 10 to 15% of patients with dry AMD are characterized by choroidal neovascularization (CNV) that leads to visual distortions and then vision loss [12,13]. In wet AMD, senescent hRPE cells produce not only excessive amounts of pro-inflammatory cytokines as in dry AMD but also vascular endothelial growth factor (VEGF) [14] and angiopoietin-2 (Ang-2) [15], which are induced by the chemotactic substances accumulated in drusen deposits. Indeed, VEGF contributes to the breakdown of the blood–retinal barrier [16] and the sprouting of fragile blood vessels, which leak their content into the layers of the retina [16], thus significant macular edema and exudates occur [12]. The integrity of the blood–retinal barrier and the maintenance of hRPE cell polarity rely on the expression and membrane localization of tight junction (TJ) proteins such as Zonula Occludens (ZO-1) or the Clostridium perfringens enterotoxin receptor (Claudin-4), as well as adherens junction (AJs) proteins such Vascular Endothelial Cadherin (VE-cadherin, Cadherin-5, or CD144) or Epithelial cadherin (E-cadherin or Cadherin-1) [17,18]. Prolonged inflammation, associated with chronic age-related disorders [19], and angiogenesis, stimulated by injured, diseased, or hypoxic tissues [20], are key factors in AMD ocular pathology [21]. Unfortunately, there are no established treatments to prevent the development of AMD, and the only treatment applied in the clinical management of wet AMD targets is VEGF [22], although with some complications. In this scenario, the importance of investigating new combinatorial anti-inflammatory and anti-angiogenic treatments could be a promising approach to AMD disease.

Vitamin D (VD, the active form 1,25-dihydroxyvitamin D3) has been shown to play a significant role in preserving the integrity of the blood–retinal barrier [23]. Ocular barrier epithelial cells can produce active vitamin D and are sensitive to the hormone [24]. In vitro and in vivo experiments showed that vitamin D inhibits the angiogenic process in a dose-dependent manner [25]. Moreover, several studies have described its anti-inflammatory and antioxidant effects on retinal pigment epithelial and endothelial cells [26,27]. Cross-sectional studies suggest that vitamin D may exert a protective effect on AMD formation; in fact, the correlation between serum vitamin D levels and AMD has been described in several studies [28], with discrepancies suggesting that vitamin D efficacy is limited by individual sensitivity [29] or may only work in a specific population [28]. Consequently, the potentiation of vitamin D efficacy, possibly by association with other natural compounds, would be highly desirable.

Among nutrients that exert an epigenetic control and can modify the expression of critical genes associated with physiologic and pathologic processes [30], sulforaphane is a natural epigenetic modulator of the vitamin D receptor [31]. Sulforaphane (SF, 1-isothiocyanato-4-methyl sulfonyl methane), mainly found in cruciferous vegetables, is known to exert anti-inflammatory effects and ameliorate injury and inflammation in hRPE cells caused by white and blue light, respectively [17], and alleviate lipopolysaccharides (LPS)-induced inflammatory injury in ARPE-19 cells by repressing the PWRN2/NF-kB (Prader–Willi Region Non-Protein Coding RNA 2/nuclear transcription factor-kappa B) pathway [32]. In addition, SF has been shown to inhibit hypoxia-inducible factor alpha (HIF-1α) and VEGF expression and migration in human colon cancer cells [33], exert anti-angiogenic effects against hepatocellular carcinoma through the inhibition of STAT3 (signal transducer and activator of transcription 3)/HIF-1α/VEGF signaling [34], and block TGF-β-induced migration in retinal endothelial cells [35]. 

The aim of this study was to investigate the synergistic effects of a supplementation of vitamin D and sulforaphane as a complementary treatment to decrease the pro-inflammatory and pro-angiogenic stimulation of epithelial cells, which contribute to the development of AMD in vivo. A retinal epithelial cell culture model (ARPE-19 cell line) was investigated in vitro after stimulation with transforming growth factor-beta (TGF-β), a pro-inflammatory cytokine active on RPE cells and produced by the cells themselves. This model of an inflamed retinal epithelium was treated with vitamin D and sulforaphane to assess their effectiveness in abating the intracellular events that promote AMD.

## 2. Results

### 2.1. Sulforaphane Potentiates the Antioxidant Activity of Vitamin D on TGF-β Dependent ROS Production

**Figure 1:**
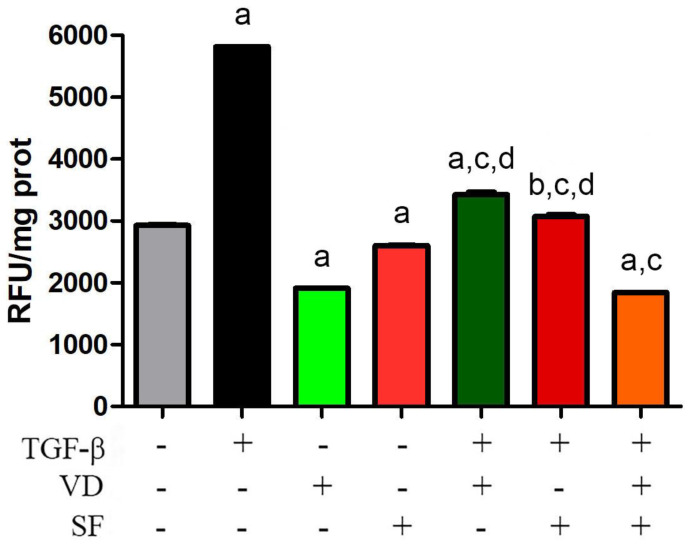
TGF-β-dependent ROS production. ROS were evaluated by fluorescence assay in untreated cells or after treatment with TGF-β, vitamin D (VD), and sulforaphane (SF) alone and in combination. Histogram values represent the mean ± SEM. The results were normalized for protein content and are expressed as fluorescence units (RFU)/mg protein. Significance of the results was evaluated vs. untreated: ^a^ *p* < 0.001 and ^b^ *p* < 0.01; vs. TGF-β−treated: ^c^ *p* < 0.001; vs. TGF-β + VD + SF: ^d^ *p* < 0.001.

It is known that the TGF-β signaling pathway is an important component of oxidative stress-induced damage [36]. In fact, TGF-β and ROS induce each other and create a vicious circle that is implicated in the pathogenesis of many human diseases; indeed, it is not easy to find a disorder without ROS in its origination and development [37]. In the case of AMD, oxidative stress-related damage to RPE is considered not only an early event in macular degeneration induction but also in the progression of the disease. Therefore, reducing the level of ROS is extremely important in AMD. In this study, to test a potential ROS-targeting treatment and reduce the oxidative stress of RPE, vitamin D and sulforaphane were tested in ARPE-19 cells to curtail TGF-β activity, a known inducer of ROS production and ROS-mediated injury in these cells [38]. As shown in Figure 1, the abatement of oxidative stress was achieved by treating the retinal epithelial cells with VD and SF as antioxidant supplements. The two molecules decreased the basal production of ROS and were effective in compensating ROS production triggered by TGF-β stimulation. Moreover, the combinatorial treatment of VD together with SF potentiated the single effect of these molecules to significantly reduce TGF-β-induced oxidative stress. In fact, a single treatment with VD decreased ROS production to 59% of what was stimulated by TGF-β, but the combination of VD and SF abated ROS to 31%. 

### 2.2. Vitamin D and Sulforaphane Decrease the Respiratory Burst Triggered by TGF-β Stimulation

Many studies indicate mitochondria as a source of high levels of ROS in AMD [37,39]. This overproduction of ROS can be considered both a cause and a consequence of mitochondrial dysfunction. In any case, the maintenance of low levels of mitochondrial ROS is essential for preserving mitochondrial function, as oxidative phosphorylation (OXPHOS) can serve as an important ROS source [40] and can be damaged by oxidative stress; thus the analysis of mitochondrial function is fundamental in the pathogenesis of disorders linked to ROS generation, such as in AMD.

**Figure 2:**
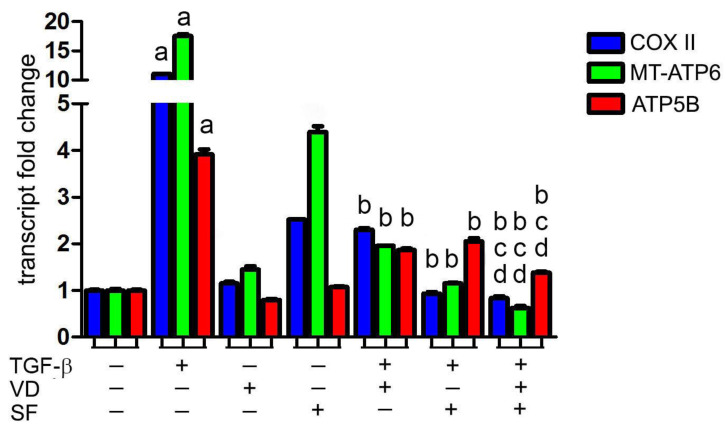
Evaluation of transcription of mitochondrial elements. The expression of cytochrome c oxidase subunit II (COX II), mitochondrially encoded ATP synthase membrane subunit 6 (MT-ATP6), and ATP synthase F1 subunit beta (ATP5B) was evaluated after combined treatment of vitamin D (VD) and sulforaphane (SF) in the presence of TGF-β. Results were normalized against the control group and expressed as transcript fold change. Significance of the results was evaluated vs. untreated: ^a^ *p* < 0.001; vs. TGF-β: ^b^ *p* < 0.001; vs. TGF-β + VD: ^c^ *p* < 0.01; vs. TGF-β + SF: ^d^ *p* < 0.01.

Based on the evidence that ROS levels are modulated by sulforaphane and vitamin D co-treatment, we investigated whether these molecules controlled mitochondrial respiratory activity during TGF-β stimulation. Indeed, this cytokine is a potent inducer of the expression of the electron transport chain components involved in oxidative phosphorylation and ROS production [41]. The expression of cytochrome c oxidase subunit II (COX II) and ATP synthase membrane subunit 6 (MT-ATP6), the two respiratory units encoded by mitochondrial DNA, and the transcription of nuclear ATP synthase F1 subunit beta (ATP5B) were investigated using real-time analysis after the stimulation of cells by TGF-β, in the presence of VD and SF alone or in combination. The RT-PCR results of respiratory elements are shown in Figure 2. Compared to the control, cells stimulated by TGF-β showed a significantly higher expression of COX II, MT-ATP6, and ATP5B mRNA levels, suggesting a respiratory burst induced by TGF-β. Notably, treatment with VD and SF alone decreased the expression of the investigated respiratory elements after TGF-β incubation, and the combined treatment potentiated the effects of the single molecules.

### 2.3. Vitamin D and Sulforaphane Reduce the Transcription Levels of IL-6 and IL-8 Induced by TGF-β

Cytokines produced by injured RPE cells may function as important regulators of intraocular inflammation [42]. When stimulated with a pro-inflammatory cytokine, such as TGF-β, RPE cells have been shown, in an in vitro model, to produce interleukin-6 (IL-6) [42,43] and interleukin-8 (IL-8) [44]. Indeed, TGF-β is also capable of enhancing or downregulating an inflammatory response depending on the cell type. 

**Figure 3:**
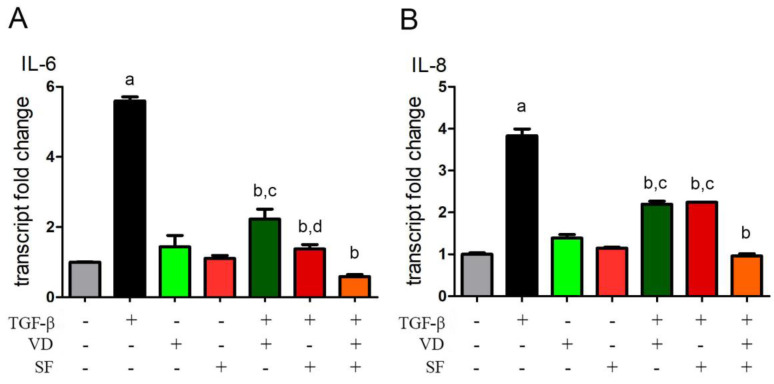
Evaluation of transcription of inflammatory cytokines IL-6 and IL-8. Quantitative PCR was carried out in TGF-β-stimulated cells to detect the level of expression of IL-6 and IL-8 mRNA in presence or absence of vitamin D (VD) and sulforaphane (SF) alone or in combined treatment. Results were normalized against the control group and expressed as transcript fold change. Significance of the results was evaluated vs. untreated: ^a^ *p* < 0.001; vs. TGF-β−treated: ^b^ *p* < 0.001; vs. TGF-β + VD + SF: ^c^ *p* < 0.001 and ^d^ *p* < 0.05.

In this study, a quantitative PCR was carried out in TGF-β-stimulated ARPE-19 cells to detect the level of expression of IL-6 and IL-8 mRNA in the presence or absence of VD and SF alone or in combined treatment. The results of this analysis, as reported in Figure 3, showed that SF can strongly potentiate vitamin D activity against TGF-β-induced inflammation by lowering the transcription of IL-6 and IL-8 cytokines. In fact, single treatment with VD decreased the transcription of inflammatory cytokines to 39% and 57% of the levels of IL-6 and IL-8 messengers induced by TGF-β, respectively, and the combination of VD and SF abated mRNAs to 10% and 25%, respectively. 

### 2.4. TGF-β-Induced VEGF Secretion Is Abated by Vitamin D and Sulforaphane

Angiogenesis is frequently associated with inflammation in adult organisms [45]. Ocular angiogenesis is related to a broad spectrum of disorders such as wet age-related macular degeneration, diabetic retinopathy, retinal artery or vein occlusion, retinopathy of prematurity, neovascular glaucoma, and corneal neovascularization, secondary to infectious or inflammatory processes [46]. Also, AMD is characterized by the over-secretion of VEGF as the major player in intraocular neovascularization [22,47]. VEGF expression is also known to be promoted by TGF-β [48]. 

**Figure 4:**
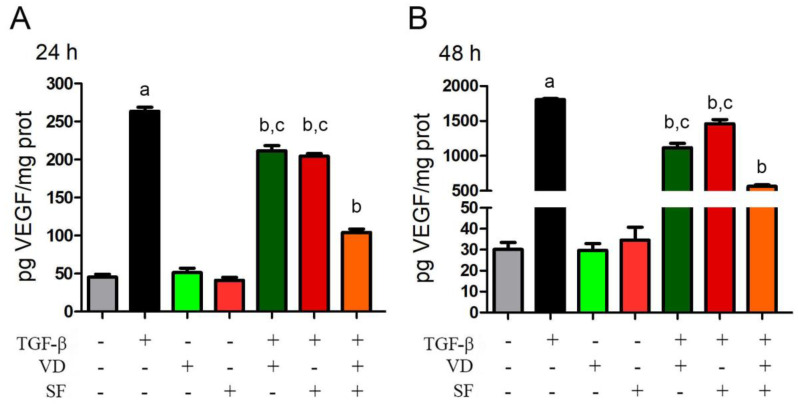
Modulation of VEGF secretion after 24 h (**A**) and 48 h (**B**). Cells were treated with TGF-β, vitamin D (VD), and sulforaphane (SF) alone and in combination. The secretion of VEGF from TGF-β−stimulated cells and its modulation by VD and SF treatments were evaluated by ELISA assay. Values were normalized for protein content and expressed as pg/mg cellular proteins. Significance of the results was evaluated vs. untreated: ^a^ *p* < 0.001; vs. TGF-β−treated: ^b^ *p* < 0.001; vs. TGF-β + VD + SF: ^c^ *p* < 0.001.

On this basis, it was interesting to evaluate the secretion of VEGF from TGF-β-stimulated ARPE-19 cells and its modulation by VD and SF treatments. The ELISA assay of secreted VEGF was conducted on cellular supernatant, and the results are shown in Figure 4. As expected, TGF-β significantly induced VEGF secretion both after 24 h and 48 h, whereas VD and SF reduced the levels of VEGF in the cellular supernatant after TGF-β stimulation, and their combination was more effective than the single compounds. In fact, after 24 h, VD alone decreased VEGF secretion to 80% of what was stimulated by TGF-β, and the combination of VD and SF strongly abated it to 39%. Furthermore, after 48 h of VD treatment, the extracellular concentration decreased to 62% of TGF-β-induced production, and, again, the effect was potentiated to 31% by co-incubation with VD and SF. In contrast, in the absence of TGF-β, VD and SF did not affect basal VEGF secretion.

### 2.5. Epithelium Integrity and Differentiation Are Enhanced by Vitamin D and Sulforaphane Treatment

It has been reported that oxidative stress causes damage to RPE cells [49] and AMD is associated with increased VEGF secretion. As VEGF disrupts ZO-1 organization, resulting in tight junction disassembly and increased monolayer permeability [50], we further investigated the expression of cell junctions in ARPE-19 cells under TGF-β stimulation. ZO-1, Claudin-4, and E-cadherin mRNA levels were measured as indicators of the integrity of junctions and the epithelium, respectively, in the presence of vitamin D and sulforaphane alone or in combination after pro-inflammatory stimulation.

**Figure 5:**
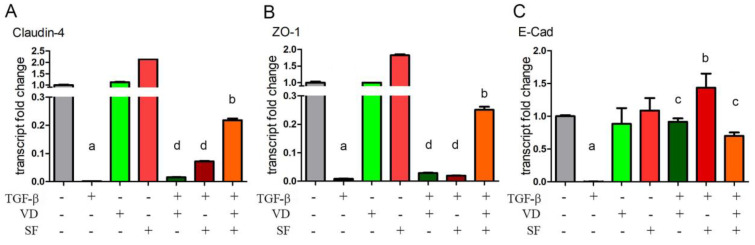
Evaluation of epithelium differentiation and integrity. Claudin-4, ZO-1, and E-cadherin mRNA levels were measured by qRT-PCR in presence of vitamin D (VD) and sulforaphane (SF) alone or in combination after TGF-β stimulation. Values were normalized against the control group and expressed as transcript fold change. Significance of the results was evaluated vs. untreated: ^a^ *p* < 0.001; vs. TGF-β−treated: ^b^ *p* < 0.001 and ^c^ *p* < 0.05; vs. TGF-β + VD + SF: ^d^ *p* < 0.001.

From the results shown in Figure 5, it is evident that TGF-β caused the disruption of cell junctions by strongly downregulating Claudin-4, ZO-1, and E-cadherin expression. The administration of VD and SF alone or, even more so, in combination, significantly restored the expression of Claudin-4 and ZO-1 inhibited by TGF-β. Also, the TGF-β-dependent abatement of E-Cadherin expression was prevented by the two compounds alone or together, although the potentiation of their effect was not evident for the combined treatment. In agreement with the results of molecular analysis, the observation of cell morphology also showed that the elongated morphology induced by TGF-β was reversed by the addition of VD and SF, as shown in Supplementary Figure S1.

### 2.6. Sulforaphane Does Not Induce VDR Expression

**Figure 6:**
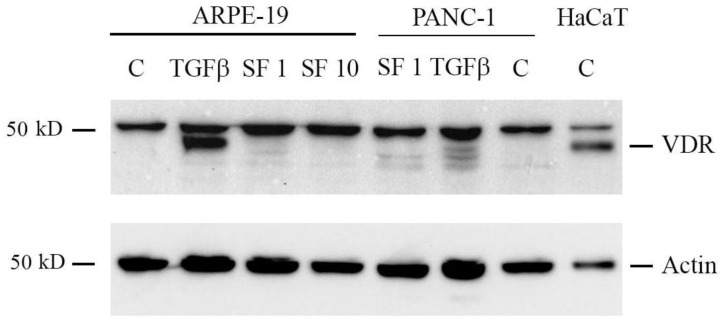
Western blot analysis of vitamin D receptor (VDR) expression. ARPE-19 and PANC-1 cells were treated with 1 or 10 μM SF and TGF-β for 48 h. Equal amounts of proteins were loaded from whole lysates obtained from ARPE-19, PANC-1, and untreated HaCaT cells (control, C), and the membrane was evaluated for the expression of VDR and actin as loading control. Original blots are shown in Supplementary Figure S2.

Since in our experimental model, we detected a potentiated effect exerted when VD and SF were used in combination, we investigated the molecular bases of this synergy. VDR is known to be epigenetically modulated by sulforaphane [31], and we hypothesized that the modulation of the receptor could be the point of convergence of the two signaling pathways. The protein expression levels of VDR in ARPE-19 cells were investigated via Western blot analysis. The correct band corresponding to VDR was identified in past studies by molecular weight and silencing experiments [51] and corresponds to the lower band when a doublet band is present, whereas the upper band is due to unspecific labeling. The signal that detects VDR is indicated in Figure 6. As a positive control for VDR expression, a whole lysate from the spontaneously transformed immortal keratinocyte HaCaT cell line was used. The results revealed that ARPE-19 cells did not express detectable basal levels of VDR since the signal corresponding to the receptor was not visible in the untreated cells (ctrl, C). However, the amount of protein was, unsurprisingly, greatly enhanced by the treatment with TGF-β since this induction has been previously described [52]. In contrast, VDR expression was not induced by SF treatment, neither at 1 µM nor 10 µM. Similarly, SF did not change VDR levels of another cell line, the pancreatic cancer cell PANC-1, as shown in Figure 6.

### 2.7. The Activity of Vitamin D and Sulforaphane Is Mediated by pERK 1/2 Signaling Pathway

Several signaling intermediates, such as MAPK/*ERK 1/2*, NF-kB, and Nrf2, could be activated in RPE-choroid AMD phenotypes and involved in the TGF-β pathway. According to preliminary research [53], they may play an important role in controlling the inflammatory response and regulating the expression of antioxidant proteins against oxidative damage, a central mechanism of pathogenesis in AMD. 

**Figure 7:**
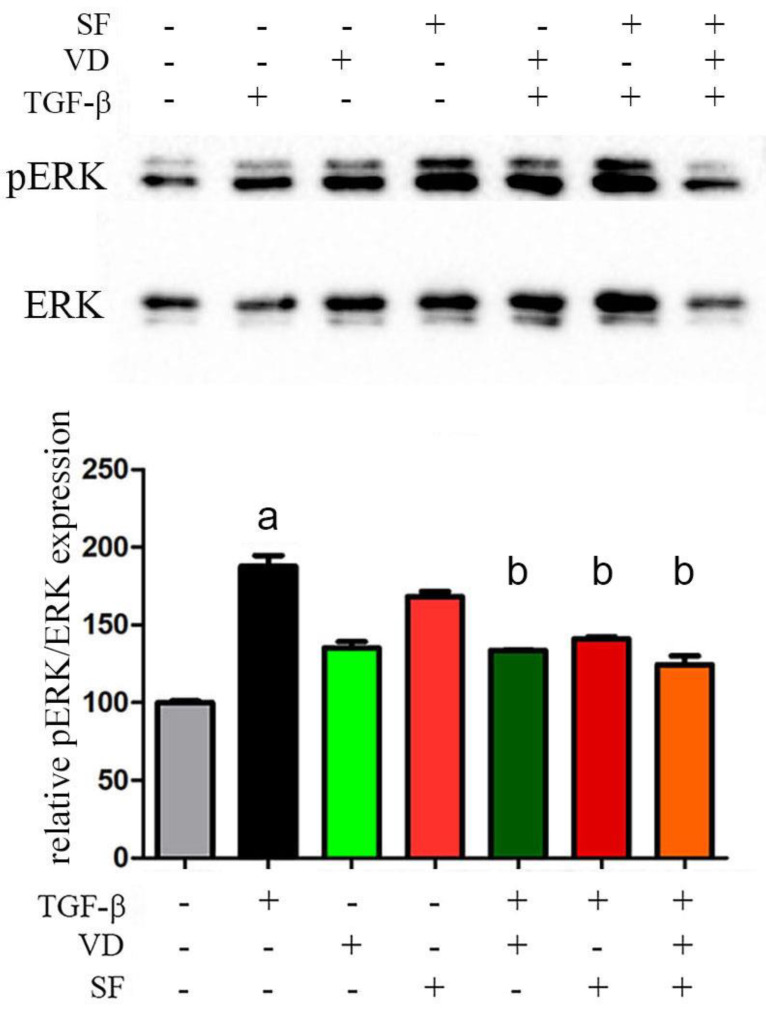
Western blot analysis of signaling pathways activation. MAPK/ERK signaling was measured as pERK/total ERK expression from whole lysates obtained in ARPE19 after treatment with TGFβ, vitamin D (VD), and sulforaphane (SF). The bands were quantified, and values were normalized against the control group (untreated) and expressed as relative pERK/ERK expression. Significance of the results was evaluated vs. untreated: ^a^ *p* < 0.001; vs. TGFβ−treated: ^b^ *p* < 0.001. Original blots are shown in Supplementary Figure S3.

As both vitamin D and sulforaphane are known to decrease inflammatory levels and reduce ROS production, it was investigated whether and which of those signaling pathways might mediate their activity. The modulation of the NF-kB and nuclear factor erythroid 2-related factor 2 (Nrf2) signaling pathways was not detected in TGF-β-treated ARPE-19 cells, while the stimulus with the inflammatory cytokine significantly triggered the activation of mitogen-activated protein kinase/extracellular signal-related kinases 1 and 2 (MAPK/ERK 1/2), measured as an increase in the phosphorylated ERK 1/2, which was hampered by the administration of vitamin D and SF alone or in combination, as shown and quantified in Figure 7. The results of this last analysis revealed that the modulation of the MAPK/ERK signaling pathway was involved in the anti-inflammatory activity of the two compounds.

## 3. Discussion

Despite the recent advances in age-related macular degeneration therapy, this disease still remains the leading cause of vision impairment for patients over 60 years of age, and its prevalence is actually increasing [54]. Currently, the few existing anti-AMD therapies can only delay or slow its progression, without providing a complete cure to patients [55]. Moreover, in patients with dry AMD, the only current treatment is antioxidant supplements and regular follow-ups to address the best corrected visual acuity (BVCA) possible [56]; on the other hand, in the wet variant, intravitreal injections of anti-VEGF agents, such as aflibercept, ranibizumab, and bevacizumab [57], are now the mainstay of treatment for age-related vascular degeneration. Faricimab, another molecule under investigation to target two of the underlying causes of AMD, both VEGF and angiopoietin-2, has been recently introduced by the Food and Drug Administration (FDA) [58]. Currently, injection frequency differs amongst physicians, which emphasizes the necessity of defining an effective strategy to handle AMD. Moreover, anti-VEGF therapy does not always result in vision acuity stabilization or improvement [59], and above all, it is not effective in ameliorating the chronic inflammatory intraocular environment, which undermines complete recovery. For this purpose, the exploitation of novel treatments based on the combination of anti-inflammatory and antioxidant agents, aiming to slow down the progression of the disease, should be taken into consideration. 

Emerging evidence has described the significant role played by TGF-β signaling in the progression of AMD [60,61]. Both TGF-β1 and TGF-β2 are produced by human RPE cells, which in turn, respond to TGF-β stimulation, but its impact remains controversial; both the inhibition and induction of TGF-β have been suggested for AMD treatment. The double role of TGF-β signaling has also been demonstrated in the axes’ activation of both classes of suppressors of mother against decapentaplegic (SMAD) proteins (TGF-β/ALK5/SMAD2/3 and TGF-β/ALK1/SMAD1/5/8), which exert opposite effects on angiogenesis and on VEGF secretion and are critical in AMD pathogenesis. Tosi et al. [61] reported both the pro- and anti-angiogenic functions of TGF-β in the late and early stages of AMD, respectively, by in vivo and in vitro experiments. Moreover, TGF-β has an immunomodulatory effect and can alter the immune reaction, participating in the inflammation process. For example, Wang et al. [60] proved that TGF-β was highly expressed in laser-induced CNV and regulated VEGF secretion, immunity, vascular fibrosis, and other processes.

ROS are abundantly generated as side products of TGF-β metabolic activity and further promote the production of the cytokine in an amplified loop that is responsible for the development of inflammation along with the subsequent secretion of VEGF. In turn, VEGF also contributes to the production of ROS, thus participating in the autostimulatory loop generated by TGF-β. In the human RPE cell line, several signaling intermediates, such as MAPK/ERK, NF-kB, and ROS, are involved in VEGF upregulation by TGF-β [62]. Based on this evidence, we set out to investigate the response of ARPE-19 cells to the cytokine and the modulatory impact of vitamin D and sulforaphane on this inflammatory stimulus. 

In recent years, the relationship between vitamin D (1,25(OH)2D) and health has received growing attention from the scientific and medical communities, as its deficiencies have been widely associated with various acute and chronic diseases. Vitamin D may also play a critical role in ocular diseases. This is supported by evidence that the vitamin D receptor and the enzymes involved in its metabolism (CYP27B1 and CYP24A1) are expressed in the retina, RPE, and choroid [27]. In addition, the anti-inflammatory properties of vitamin D in ocular disease are well known [23].

In this study, the protective effects of vitamin D have been investigated in vitro by testing the human RPE cell line, ARPE-19, and the results supported the beneficial activity of the hormone in preventing the development of the inflammatory response. In previous studies, we demonstrated the negative feedback regulation exerted by vitamin D/VDR on TGF-β activity [41], and the results of the present investigation confirm the general role of vitamin D in checking the pro-inflammatory activity of the cytokine.

In addition to vitamin D, sulforaphane could also exert a protective effect against oxidative stress in numerous biological settings. Wang et al. [63] demonstrated that SF decreased cadmium-induced ROS generation in the human bronchial epithelial cell line BEAS-2BR. Moreover, it has been shown in vivo that SF exhibits antioxidant activity by attenuating ROS and can alleviate symptoms of chronic inflammatory diseases [64]. These also include ocular diseases, where the protective effects of SF against the optical damage of RPE and photoreceptor cell degeneration have been demonstrated [65]. Interestingly, it was hypothesized that SF could also operate as a detoxifying agent against TGF-β-driven ROS production; in fact, SF has been described as a potent inducer of a family of Phase 2 detoxification enzymes and a stimulator of glutathione (GSH) biosynthesis [66]. Overall, these studies suggest the potential activity of SF in neutralizing ROS and attenuating the progression of inflammation. 

In addition, in vitro studies [67] demonstrate that SF can modulate and mitigate inflammation both by transcriptional control and also through epigenetic mechanisms. In fact, it can trigger several epigenetic alterations by modulating the histone modifications; it is of note that via the inhibition of histone deacetylase (HDAC) and the increased acetylation of histones H3 and H4, SF can induce the expression of VDR, with the consequent regulation of the antimicrobial human Beta Defensin-2 (hBD-2) peptide [68]. However, in this study on the ARPE-19 cell model, SF was not active as an epigenetic modulator of VDR; in contrast, TGF-β increased the expression of the receptor. Based on the latter observation, it is conceivable that the efficacy of vitamin D in preventing the inflammatory effects of TGF-β is mediated by upregulated VDR. 

In this study, the potentiation between the signaling pathways of vitamin D and sulforaphane has been demonstrated mainly by the control of ROS levels, associated with the modulation of mitochondrial respiration, which leads, on the one hand, to a decrease in inflammation markers, and on the other hand, to the control of VEGF expression. 

As oxidative stress is reduced, the integrity of the epithelium is also conserved. The structure of Tight Junctions plays a critical role in tissue barriers, host defense, and inflammation. TJ protein complexes are composed of integral membrane proteins, cytoplasmic plaque proteins, and cytoskeletal proteins. Among these, the claudin family membrane proteins are key components for the structure and function of TJs. The molecular composition of TJs also involves occludin and members of the zonula occludens protein family, such as ZO-1 [69]. Vitamin D is a well-known inducer of ZO-1 expression and translocation to the plasma membrane and enhancer of the epithelial marker E-Cadherin [70]. In our experimental inflammatory model, we detected the reversion of the inhibitory role of TGF-β on Claudin-4, ZO-1, and E-Cadherin when epithelial cells were treated with VD or SF. Most interestingly, the beneficial effect on TJ proteins was potentiated by the co-incubation of the two compounds, in agreement with the other experimental results of this work. 

Since the two molecules were effective and even potentiated in the response to TGF-β, a point of convergence between their signaling pathways could exist. In our cellular model, we could not detect TGF-β-dependent modulation of the NF-kB nuclear translocation, but significant activation of MAPK/ERK was triggered by the inflammatory cytokine and was reduced by the addition of VD and SF, suggesting that the modulation of this signaling pathway was involved in the anti-inflammatory activity of the two compounds. This observation is well-supported by the knowledge that oxidative stress activates MAPK signaling [71]; however, the potentiation of their inhibitory effects on MAPK/ERK activation was not clearly demonstrated, leading to the conclusion that some additional mechanism could reinforce the additive effects reported in this study. New insights into the crosstalk between MAPK/ERK and TGF-β signaling may reveal the molecular mechanism by which ERK 1/2 is activated to its phosphorylated form and explain how this may mediate the activity of vitamin D and sulforaphane. In fact, it is still under investigation whether the enhancement of VD activity by SF can result in a synergistic effect mediated by other molecular mechanisms and not just an additive one, as shown by the analysis of pERK. It is of note that Nrf2 activation was not detected in our experimental inflammatory model; therefore, the effect of SF on this pathway was not investigated, although Nrf2 may regulate the expression of antioxidant proteins.

Overall, vitamin D and sulforaphane exerted positive effects on the ARPE-19 cell model by reducing ROS production, attenuating inflammation and VEGF secretion, and thus maintaining retinal epithelium integrity. Because our in vitro inflammatory model replicates several features of the human disorder described as AMD, the positive impact of these treatments can be also expected in vivo and could be proposed as a novel strategy to further improve the quality of life of AMD patients. 

## 4. Materials and Methods

### 4.1. Cell Culture and Treatments

Spontaneously arising human retinal pigment epithelium (ARPE-19) cells and pancreatic cancer cell line PANC-1 were purchased from the American Type Culture Collection (ATCC, Manassas, VA, USA) and were cultured in Dulbecco’s modified Eagles medium supplemented with 10% fetal bovine serum and 1% antibiotics (penicillin-streptomycin) at 37 °C in a humidified 5% CO_2_ atmosphere. ARPE-19 cells were treated with 1 µM and 10 µM sulforaphane (SF) for 24 h in a single treatment or co-treatment in the absence or presence of 10 ng/mL of transforming growth factor beta (TGF-β) and/or 10 nM Vitamin D (VD). SF was obtained from Kura Srl (Torino, Italy). All reagents were purchased from Merck (Milan, Italy) and, unless otherwise specified, plasticware was from Falcon (Becton Dickinson, Franklin Lakes, NJ, USA).

### 4.2. Western Blotting Analysis

After treatments, cells were scraped from the culture dishes and homogenized in lysis buffer containing 10 nM Tris-HCl at pH 7.4, 0.33 M saccharose, 1 mM dithiothreitol, 1 mM EDTA, 1 mM proteases inhibitor Cocktail set III (Calbiochem), 1 mM phenylmethylsulphonyl fluoride (PMSF), 1 nM Na_3_VO_3_, and 1 mM NaF. Lysates were subjected to differential centrifugation to isolate the nuclear and mitochondrial fractions, as previously described [41,52,72]. Western blot analysis of 50 µg of total lysate, of mitochondrial and nuclear fractions, was carried out after separation by 10% or 12% SDS-PAGE and transferred to a polyvinylidene fluoride (PVDF) membrane. Mouse monoclonal antibody anti-VDR (sc-13133, Santa Cruz Biotechnology Inc., Santa Cruz, CA, USA), rabbit anti-ERK 1/2 (C-9; sc-514302, Santa Cruz Biotechnology Inc., Santa Cruz, CA, USA), and rabbit anti-phospho ERK 1/2 (12 D-4; sc-81492 Santa Cruz Biotechnology Inc., Santa Cruz, CA, USA) were used to detect the proteins of interest. The loading controls were checked on the same membranes and detected by antibody anti-actin (mouse monoclonal sc-8432, Santa Cruz Biotechnology Inc., Santa Cruz, CA, USA), which was used as a reference to correct the quantification of the proteins in total, mitochondrial, and nuclear fractions, respectively. After overnight incubation, the membrane was washed with 0.1% *v*/*v* PBS-Tween and subjected for 1 h to a peroxidase-conjugated anti-mouse or anti-rabbit secondary antibody (diluted 1:5000 *v*/*v* in 5% *w*/*v* PBS-Tween with milk, Bio-Rad Laboratories, Hercules, CA, USA). 

The correct band corresponding to VDR was identified in past studies using molecular weight and silencing experiments [51,72], and it corresponds to the lower band when a doublet band is present, whereas the upper band is due to unspecific labeling. The signal that detects VDR is indicated in the figure. 

Protein electrophoresis bands were quantified using scanning digital densitometry with ImageJ software analysis (ImageJ version 1.29, Sun Microsystems Inc., Palo Alto, CA, USA). All data were expressed as mean ± S.D of three independent experiments.

### 4.3. Measurement of Intracellular ROS production

After treatments, ARPE-19 cells were harvested and loaded with 10 M 2′,7’-dichlorodihydrofluorescein diacetate (DCFH-DA, Merck, Milan, Italy) for 15 min, as previously reported [73]. The fluorescence values were expressed as relative fluorescence units (RFUs) and were normalized to the protein content.

### 4.4. Real-Time Polymerase Chain Reaction (qRT-PCR)

Total RNA was extracted with TRIzol^®^ (Invitrogen, Thermo Fisher Scientific, Waltham, MA, USA) after treatments. One microgram of total RNA was reverse-transcribed into cDNA using an iScript cDNA Synthesis Kit (Bio-Rad Laboratories AG, Cressier FR, Switzerland) according to the manufacturer’s instructions. The RT-PCR primers were designed with NCBI/Primer-BLAST and synthesized by Merck (Milan, Italy). Quantitative PCR was carried out in a final volume of 25 µL using the IQ™ SYBR Green Supermix (Bio-Rad Laboratories AG, Cressier FR, Switzerland) with specific primers for the quantitation of the following human genes: cytochrome c oxidase subunit 2 (COXII; fwd 5′–TCTGGTCAGCCCAACTCTCT–3′, rev 5′–CCTGTGATCCACCAGAAGGT–3′, mitochondrial ATP synthase F0 subunit 6 (MT-ATP6; fwd 5′–CCAATAGCCCTGGCCGTAC–3′, rev 5′–CGCTTCCAATTAGGTGCATGA–3′), mitochondrial ATP synthase F1 β subunit (ATP5B, fwd 5′-GTGGGCTATCAGCCTACCCT-3′, rev 5′-CAAGTCATCAGCAGGCACAT-3′ [74], interleukin-6 (IL-6, fwd 5′-GGTACATCCTCGACGGCATCT-3′, rev 5′-GTGCCTCTTTGCTGCTTTCAC-3′) [73], interleukin-8 (IL-8, fwd 5′-GGAGAAGTTTTTGAAGAGGGCTGA-3′, rev 5′-TGCTTGAAGTTTCACTGGCATCTT-3′), the junction adhesion and stent protein zonula occluden-1 (ZO-1, fwd 5′-AAAGAGAAAGGTGAAACACTGC-3′, rev 5′-TTTTAGAGCAAAAGACCAACCG-3′) [18], E-cadherin (E-cadherin, fwd 5-′TACGCCTGGGACTCCACCTA-3′, rev 5′-CCAGAAACGGAGGCCTGAT-3′) and closure protein-4 (claudin 4, CLDN4; 5‘ CCACAACATCATCCAAGACTTC-3′, rev 5′- CAGAATACTTGGCGGAGTAAGG-3′ [75]. PCR amplification was conducted with 1 cycle of denaturation at 95° C for 2 min, 45 cycles of amplification including denaturation at 95° C for 15 s, and annealing/extension at 60° C for 30 s. Data were analyzed using the 2−DDCT, and the housekeeping gene beta-2 microglobulin (β2M, fwd 5′–AGCAAGGACTGGTCTTTCTATCTC–3′, rev 5′–ATGTCTCGATCCCACTTAACTA–3′) was used as the reference gene to normalize the cDNA in different samples. Nonspecific amplifications were not detected, and the specificity of PCRs was confirmed using melt curve analysis.

### 4.5. Vascular Endothelial Growth Factor (VEGF) Quantification

After treatments, the concentrations of the vascular endothelial growth factor (VEGF) were determined in culture supernatants using an enzyme-linked immunosorbent assay, according to the manufacturer’s protocol (R&D Systems, Inc., Minneapolis, MN, USA). Analyte concentrations in supernatants were expressed as pg/mg cellular proteins.

### 4.6. Statistical Analysis

Statistical analysis of data was performed using the ANOVA test with Tukey’s post-hoc correction, using GraphPad Prism 9.0 (GraphPad Software, Inc., San Diego, CA, USA). *p* values < 0.05 were considered significant. All data are expressed as the mean S.E.M. of three independent experiments.

## 5. Conclusions

This study demonstrated that the beneficial effects of vitamin D to soothe inflammation, decrease VEGF secretion, and restore epithelial integrity were potentiated by SF. 

Based on this evidence, the combination of the two compounds could be proposed as a preventive approach and/or in association with anti-VEGF treatment to counteract the chronic oxidative stress at the basis of the development of AMD. Indeed, by abating the inflammation that participates in the pathogenesis of AMD, the association of these two molecules could be exploited in treatment plans in terms of reducing the doses of serial intravitreal injections and follow-up visits for patients, hopefully increasing their quality of life. 

## Data Availability

All data generated or analyzed during this study are included in this published article.

## Supplementary Materials

The following supporting information can be downloaded at: https://www.mdpi.com/article/10.3390/ijms25126404/s1.
