# Supplementary figures and images for: Vitamin D and Sulforaphane Decrease Inflammatory Oxidative Stress and Restore the Markers of Epithelial Integrity in an In Vitro Model of Age-Related Macular Degeneration
Source: Int J Mol Sci. 2024 Jun 10;25(12):6404. doi: 10.3390/ijms25126404 (PMC11203625; doi:10.3390/ijms25126404)

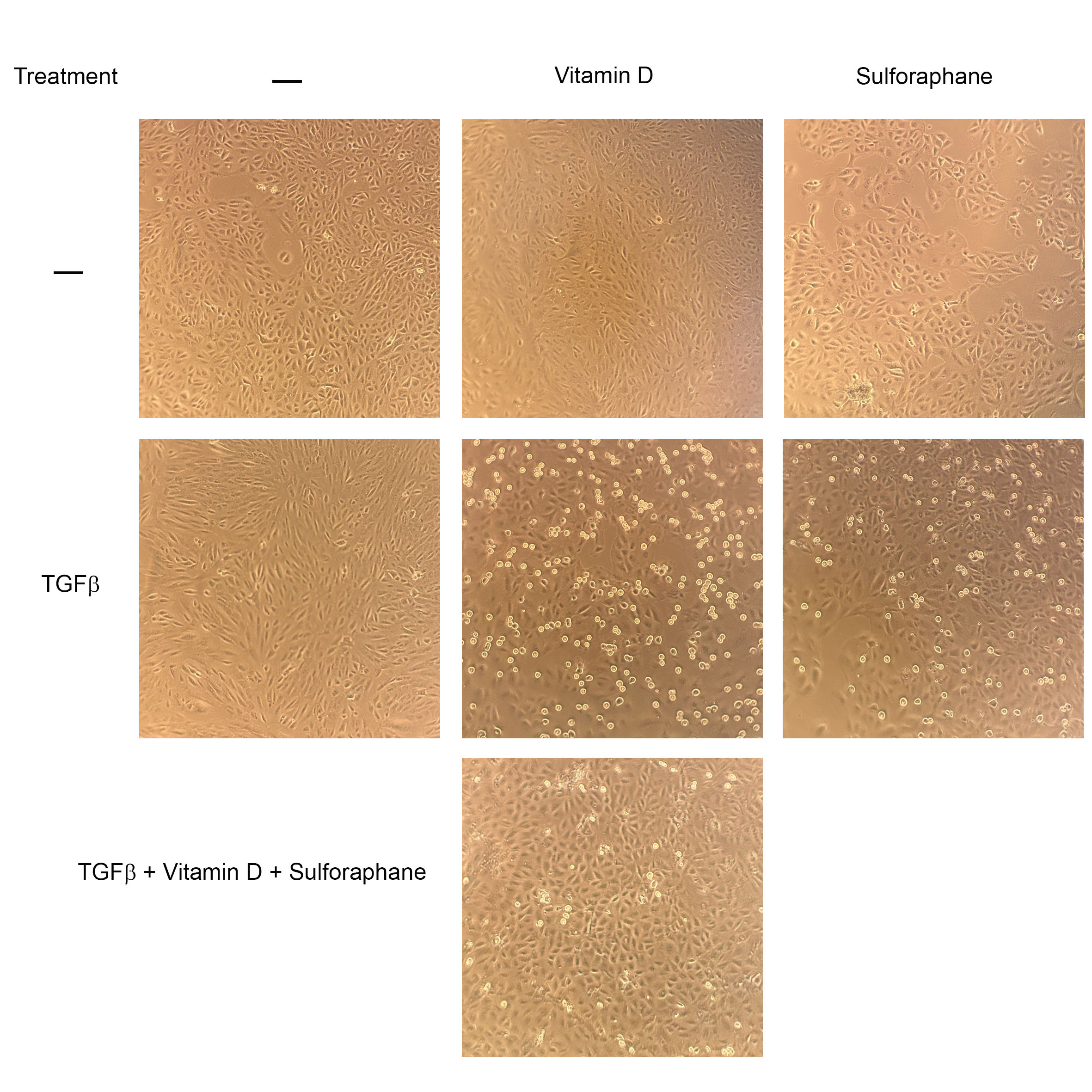

Supplement: Supplementary file 1 [file ijms-25-06404-s001.zip › Figure S1.jpg]

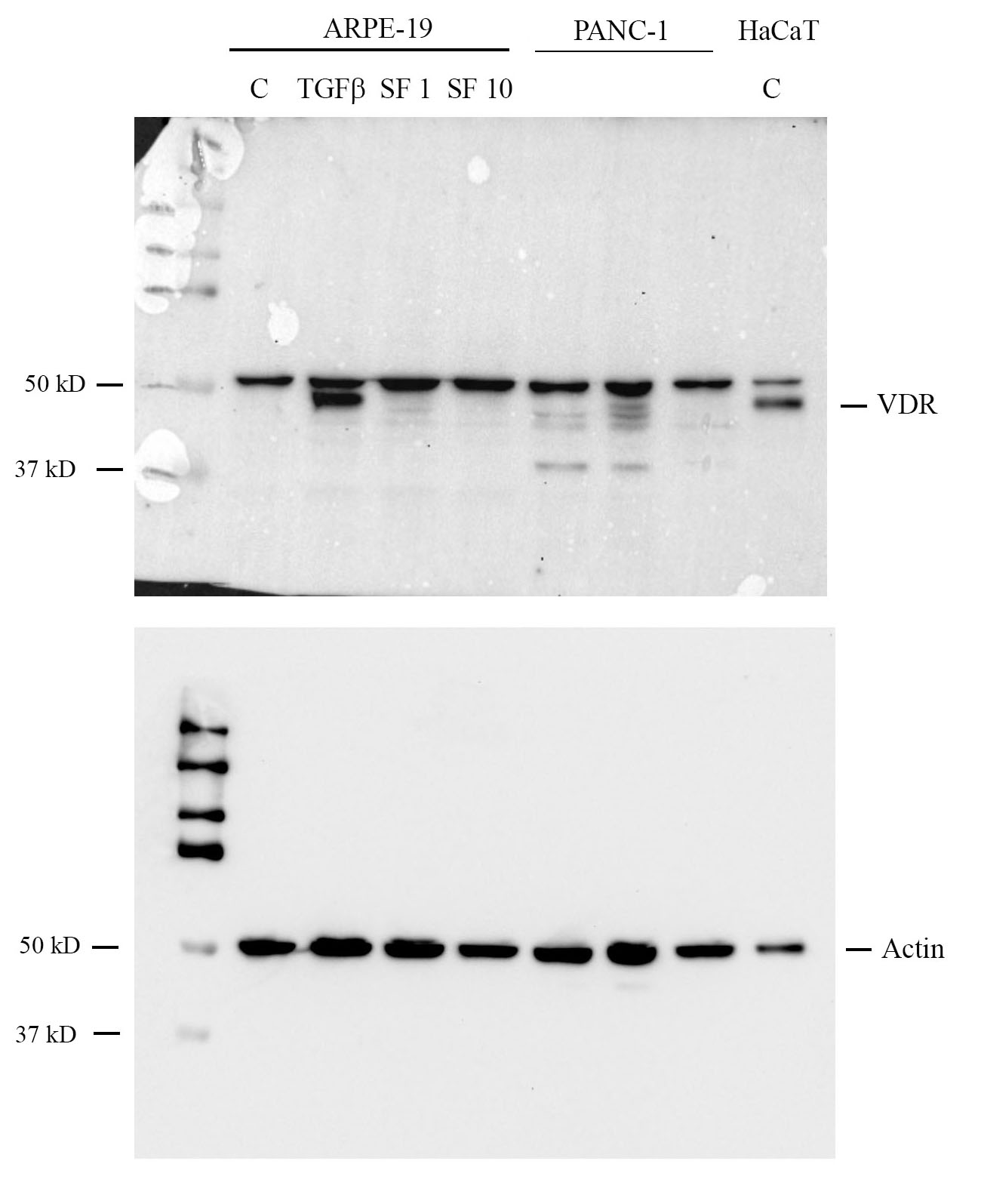

Supplement: Supplementary file 1 [file ijms-25-06404-s001.zip › Figure S2.jpg]

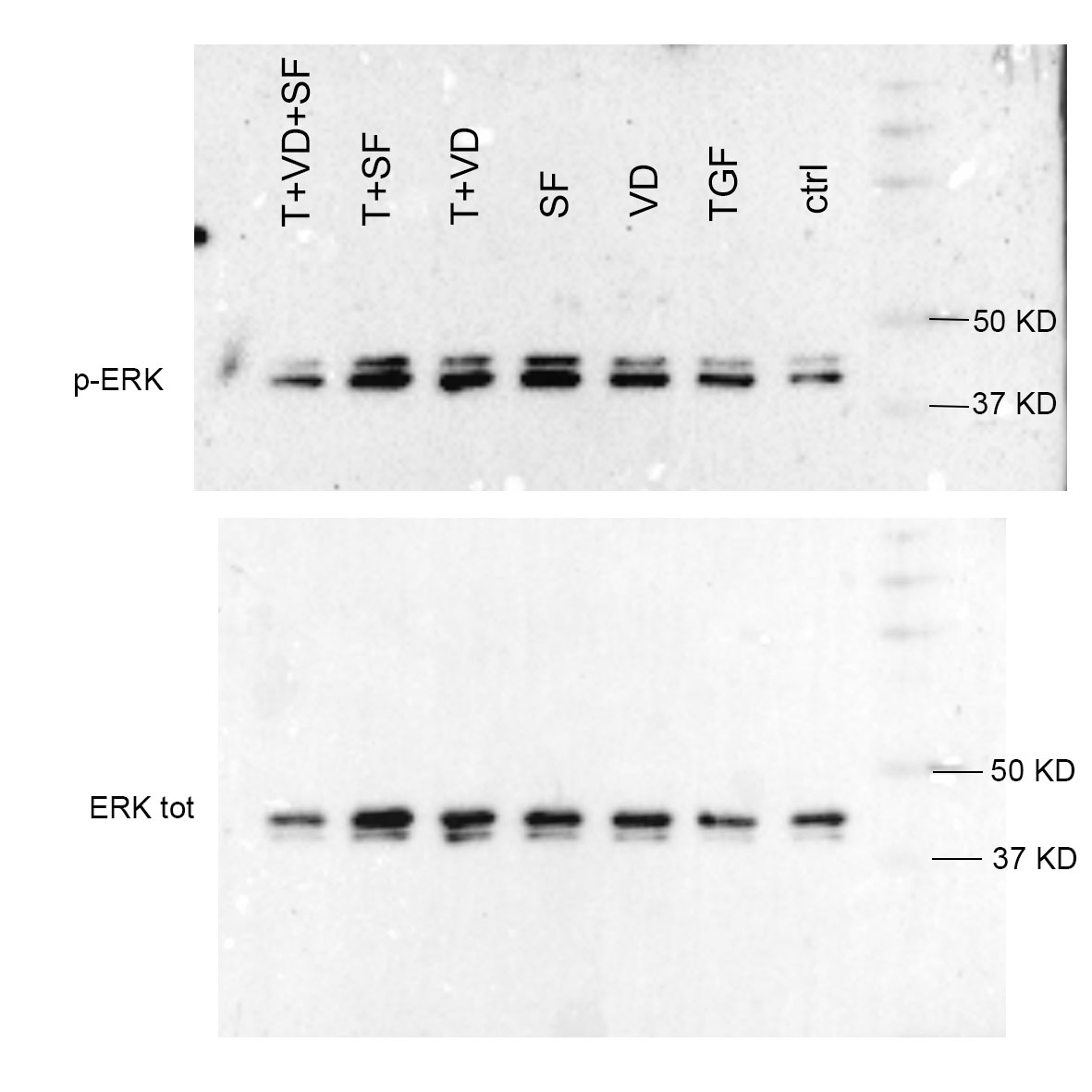

Supplement: Supplementary file 1 [file ijms-25-06404-s001.zip › Figure S3.jpg]
